# Reproduction of *Meloidogyne arenaria* race 2 on Flue-cured tobacco possessing resistance genes Rk1 and/or Rk2

**DOI:** 10.21307/jofnem-2021-042

**Published:** 2021-04-01

**Authors:** Noah Adamo, Charles S. Johnson, T. David Reed, Jonathan D. Eisenback

**Affiliations:** Virginia Tech, Southern Piedmont Agricultural Research and Extension Center, 2375 Darvills Rd, Blackstone, VA 23824; Virginia Tech, School of Plant and Environmental Sciences, 170 Drillfield Dr, Blacksburg, VA 24060

**Keywords:** *Meloidogyne arenaria*, *Nicotiana tabacum*, Flue-cured tobacco, Resistance

## Abstract

Resistance to *Meloidogyne incognita* races 1 and 3 and race 1 of *M. arenaria* is imparted to flue-cured tobacco by the gene *Rk1. Meloidogyne arenaria* race 2 is not controlled by *Rk1* and has become prevalent in Virginia. A second form of resistance effective against *M. javanica*, *Rk2*, is also increasingly available commercially. Greenhouse and field trials including a root-knot susceptible cultivar, cultivars homozygous for *Rk1* or *Rk2*, and cultivars possessing both genes were conducted in 2018 and 2019 to investigate the effect of *Rk1* and/or *Rk2* on parasitism and reproduction of *M. arenaria* race 2. Plants were inoculated with 5,000 *M. arenaria* race 2 eggs in the greenhouse or infested by a native nematode population in the field. Data were collected after 28 days (greenhouse) or every 3 weeks following transplant until 18 weeks in the field and included root galling index, nematodes present in roots, egg mass numbers, and egg counts; reproductive indices were also calculated. We found that the combination of *Rk1* and *Rk2* provides greater resistance to *M. arenaria* race 2 than either gene alone. While the effect of either gene alone was inconsistent, we did observe some significant reductions in galling and reproduction associated with each gene relative to the susceptible control.

Tobacco (*Nicotiana tabacum* L.) is a valuable agricultural commodity, cultivated around the world (FAO, 2016). While tobacco production in the United States has decreased over the past two decades, the crop still contributed over 1.2 billion dollars to the economy in 2016 (FAO, 2016). Flue-cured tobacco accounts for the majority of tobacco production in the United States, with over 14,000 acres planted in Virginia in 2020 (USDA, 2020). Root-knot nematodes (*Meloidogyne* spp.) can cause serious issues for flue-cured tobacco growers in the Southeastern United States, and may reduce yields by 1 to 5% in Virginia (Fortnum et al., 2001; Koenning et al., 1999). In the past several decades, some of the most effective chemical controls for root-knot nematodes have become unavailable to tobacco growers (LaMondia, 2008; USEPA, 2008). As such, the use of root-knot resistant or tolerant cultivars is an essential tool for root-knot nematode management in flue-cured tobacco (Johnson et al., 2005).

Nematode resistance is defined as the inhibition of reproduction by a nematode on a given host (Roberts, 2002). On nematode tolerant hosts, nematode reproduction is not necessarily inhibited, but tolerant hosts do not exhibit adverse responses to nematode parasitism in aspects such as vigor and yield (Roberts, 2002). Root-knot nematode resistance was first introduced into a commercial tobacco cultivar in 1961 in the form of the gene *Rk*, know referred to as *Rk1*, which was originally discovered in *N. tomentosa* Ruis and Pav. (Yi et al., 1998). This gene has been widely incorporated into flue-cured tobacco cultivars grown commercially in the United States (Koenning et al., 1999). *Rk1* imparts resistance to *M. incognita* (Kofoid and White, 1919) Chitwood (1949) host races 1 and 3 and *M. arenaria* (Neal, 1889) Chitwood (1949) host race 1 (Ng’ambi et al., 1999b; Schneider, 1991). Ternouth et al. (1986) suggested that the gene imparts some level of resistance or tolerance to *M. javanica* (Treub, 1885). However, Ng’ambi et al. (1999b) reported that *Rk1* imparts little or no resistance to *M. javanica*, *M. incognita* host races 2 and 4, *M. arenaria* race 2, and *M. hapla* Chitwood 1949.

Another gene, originally known as ‘*T*,’ was discovered in Zimbabwe in 1950 (Schweppenhauser, 1975). This gene was present in *N. tabacum* plants in subsistence gardens along the Zambezi River that had been planted continuously for over 250 years in soils heavily infested with *M. javanica* (Mackenzie et al., 1986; Schweppenhauser, 1975; Ternouth et al., 1986). Individual plants exhibiting what was termed a partial resistance to *M. javanica* did not support reproduction and had only limited development of adult female nematodes in preliminary experiments (Schweppenhauser, 1975). Subsequent research suggested that ‘*T*,’ or *Rk2* as it is also known, conferred a higher level of resistance to *M. javanica* than *Rk1*, also known in Zimbabwe and South Africa as ‘*S*’ (Ternouth et al., 1986). This research also demonstrated that ‘stacking’ both genes in a plant selection induced a very high level of resistance to *M. javanica* (Ternouth et al., 1986). In addition, a 1982 report (Shepherd) referred to significant reductions in successful root penetration by juveniles of *M. javanica* on ‘better breeding lines’ relative to susceptible entries. It may be reasonably inferred that these ‘better breeding lines’ were selections carrying the *Rk2*, or ‘*T*’ gene. If this is the case, the implied mechanism of resistance associated with *Rk2* or ‘*T*’ would be considerably different from that associated with *Rk1*, which does not reduce root penetration by juveniles, but inhibits formation of giant cells (Schneider, 1991). Schweppenhauser et al. (1975) had originally suggested that ‘*T*’ was in effect a quantitative trait locus, but ultimately concluded that ‘*T*’ or *Rk2* is a monogenic, dominant gene with effects augmented by one or two additional genes.

In the 1980s, Zimbabwean researchers crossed local plant selections possessing the ‘*T*’ gene with established entries to improve agronomic traits that were lacking in the landrace tobacco selections (Mackenzie et al., 1986). The resulting line, RKT15-1-1, was crossed subsequently with the established flue-cured tobacco cultivars SC 72 and NC 89 (Ternouth et al., 1986). This work resulted in two breeding lines, STNCA and STNCB, which possessed both *Rk1* and *Rk2*, and which were also subsequently crossed with a number of established flue-cured cultivars to refine and define the agronomic and flue-cured characteristics of the lines (Ternouth et al., 1986). The *Rk2* gene first became commercially available in Zimbabwe in 1993, when a cross between STNCB 2-28 and ms Kutsaga E1 was released, which incorporated *Rk2* into a flue-cured tobacco cultivar along with *Rk1* (Jack and Lyle, 1999; Jack, 2001; Way, 1994). Flue-cured tobacco cultivars possessing both *Rk* genes became commercially available in the United States in 2007, when a number of cultivars were released by Cross Creek Seed Company and ProfiGen do Brasil (Reed, 2007). This combination of root-knot nematode resistance has become increasingly available to growers since 2007 (Johnson, 2020)

Surveys of flue-cured tobacco fields in Virginia conducted over the past two decades have demonstrated that *M. incognita*, which has historically been the most widespread root-knot nematode species in Virginia tobacco fields (Johnson, 1989), has been superseded in prevalence by *M. arenaria* (Eisenback, 2012). In 2004, root-knot nematodes were present in 43.5% of 170 surveyed flue-cured tobacco fields, with *M. arenaria* infesting 56.7% of the fields surveyed, while *M. hapla, M. incognita,* and *M. javanica* infested 25.0, 16.7, and 11.7% of surveyed fields, respectively (Eisenback, 2012). As of 2010, the proportion of infested fields had not changed meaningfully (44.9%), with similar trends in species distribution observed in 276 surveyed Virginia tobacco fields. *Meloidogyne arenaria* continued to predominate, infesting 58.8% of surveyed fields, while *M. incognita* was less abundant, infesting 11.1% of surveyed fields (Eisenback, 2012); unidentified *Meloidogyne* species accounted for between 6.3 and 8.3% of the collected specimens in both years (Eisenback, 2012). The increased prevalence of *M. arenaria* presents a potential challenge to growers, as the *Rk1* gene is only effective in managing race 1 this species, and the resistance or tolerance conferred to flue-cured tobacco against *M. arenaria* race 2 by *Rk1* in combination with *Rk2* are unclear. While previous work in Virginia has confirmed that *Rk1* in combination with *Rk2* confers resistance to *M. javanica* (Ma and Johnson, unpublished data) and *M. incognita* race three (Pollock et al., 2016), it is crucial to better understand the impact of these genes on *M. arenaria* race 2. The presented work investigated the effect of *Rk1* and *Rk2,* alone and in combination, on the penetrative and reproductive capacity of a population of *M. arenaria* race 2 on flue-cured tobacco.

## Materials and methods

### Greenhouse trials

A population of *M. arenaria* race 2 collected from a flue-cured tobacco field in Halifax County, VA was maintained on susceptible tomato (*Solanum lycopersicum* L.) variety ‘Rutgers’ in greenhouses at the main Virginia Tech campus in Blacksburg, VA and at the Southern Piedmont Agricultural Research and Extension Center (SPAREC) near Blackstone, VA. The species identity had been previously confirmed by differential host testing (Taylor and Sasser, 1978) and was verified by examination of more than 40 perineal patterns from the population (Eisenback, 1985). Three greenhouse experiments were conducted in 2018 at SPAREC with an additional trial in 2019 at the main Virginia Tech Campus in Blacksburg, VA in order to evaluate the impact of resistance genes *Rk1* and/or *Rk2* on parasitism of flue-cured tobacco by *M. arenaria*. Experiments were arranged in randomized complete block designs with seven replications, except for the test in Blackstone in September, 2018, which had six replications. These experiments assessed resistance to *M. arenaria* in a panel of five flue-cured tobacco entries: Hicks (susceptible to the four major tropical root-knot nematode species, *M. incognita, M. javanica, M. arenaria,* and *M. hapla*); K326 (homozygous for *Rk1*); T-15-1-1 (homozygous for *Rk2*); CC 13 (homozygous for *Rk1* and heterozygous for *Rk2*); and STNCB-2-28 (homozygous for both *Rk1* and *Rk2*). Seed of K326 and CC 13 were donated by Cross Creek Seed, Raeford, NC, while seed of the remaining four entries were produced in the SPAREC flue-cured tobacco nursery in 2016 and 2017. Seed were germinated in organic vermiculite (The Epsoma Company, Millville, NJ) and four to five week old seedlings were transplanted to individual 7.6 cm clay pots containing a 2:1 mixture of steam sterilized sandy loam field soil with Profile Greens-Grade Porous Ceramic soil amendment (Profile Products, Buffalo Grove, Il). Seedlings with four to six true leaves were transplanted into 15 cm clay pots. Egg inoculum for greenhouse trials was collected from infested roots using the method of Hussey and Barker (1973). Eggs were collected in 1 L of tap water, counted with a compound microscope at X 20 to 40, and an egg suspension calibrated to contain 125 eggs/mL of suspension immediately prior to inoculation. Plants were inoculated with a 40 mL aliquot containing 5,000 nematode eggs applied directly to the root mass during transplanting. Plants were maintained in greenhouses at ambient air temperatures of 20 to 33°C, with natural lighting.

Plant roots were washed free of soil substrate 28 days following inoculation and blotted dry; the aerial portion of the plants were discarded. Fresh root weights were recorded and the whole root system was evaluated for galling according to the gall count index developed by Taylor and Sasser (1978), in which gall counts are ranked as follows: 0 galls present = 0; 1–2 galls present = 1; 3–10 galls present = 2; 11–30 galls present = 3; 31–100 galls present = 4; and more than 100 galls present = 5. Roots were cut into 4 to 6 cm long sections and thoroughly mixed. Root-knot presence in roots was assessed in three 1 g subsamples from each plant. Feeder roots were cleared in 1% sodium hypochlorite and stained with 0.005% acid fuchsin (Byrd et al., 1983). Roots were examined and nematodes were counted with a stereomicroscope at X10 to 40. For egg mass counts, two 1 g subsamples were stained in 0.15 g/L Phloxine-B (Daykin and Hussey, 1985) for approximately 5 min and were counted with a stereomicroscope at X10 to 40. Eggs were extracted from the remaining root system in the manner described above and counted at X40 using an inverted compound microscope. The reproductive index for each plant was calculated by dividing the final egg count (P_f_) by the initial egg inoculum number (P_i_).

### Field trials

Field trials were conducted in 2018 and 2019 in a flue-cured tobacco field in Palmer Springs, VA infested with a population of *M. arenaria* race 2 in order to compare the flue-cured tobacco entries described above. Trials were arranged in randomized complete block designs with 11 replications in 2018 and 10 replications in 2019. Treatments were randomized independently each year. Plots consisted of single 16.1 m long rows spaced 1.2 m apart. Plots were mechanically transplanted, fertilized, and maintained in accordance with the recommendations of Virginia Cooperative Extension (Reed et al., 2018).

Initial soil nematode population densities were estimated each year based on soil samples collected between bed formations and transplanting. Final population densities were determined after final samples have been collected 18 weeks after transplant. Twenty-four 2 cm by 16 cm soil samples were collected from each plot and bulked. Nematode counts were performed at the Virginia Tech Nematode Diagnostic and Assay Lab on the Virginia Tech Blacksburg Campus. Bulked samples were initially hand mixed to reduce aggregates, sifted, and a 250 cubic centimeter subsample from each plot was subjected to nematode extraction using a mechanical elutriator, sugar flotation, and decantation sieving (Barker, 1985). Two plants were destructively sampled from each plot beginning three weeks after transplanting, and every three weeks until 18 weeks after transplanting. Soil was washed free of the root systems of the sampled plants and galling was assessed as described above. Fresh weights of the entire root systems were recorded and fibrous feeder roots were separated from lignified structural roots. The number of nematodes present in roots, egg masses on roots, and eggs per gram of root were determined for the fibrous feeder root portion of the root system of each plant. Egg mass production was evaluated by counting Phloxine-B stained egg masses (Daykin and Hussey, 1985) on three 1 g subsamples at X10 to 40. Numbers of nematodes present in roots were determined by clearing two 1 g subsamples of feeder roots with sodium hypochlorite and staining the roots with acid fuchsin (Byrd et al., 1983) that were examined with a stereomicroscope at X10 to 40. Eggs were extracted from the remaining feeder roots by agitation in 1% sodium hypochlorite (Hussey and Barker, 1973). Eggs were suspended in 1 L of tap water and two 10 mL aliquots were counted at X40 with a compound microscope. These counts were used to calculate the approximate number of nematodes extracted from the known mass of feeder roots, which was then used to calculate the number of eggs per gram of feeder root for the entire root system.

### Statistical analysis

Data were transformed (log_10_ (*x* + 1)) prior to statistical analysis. Data from all trials were subjected to analysis of variance (ANOVA) using PROC GLM in SAS (version 9.4; SAS Institute, Cary, NC). Differences among treatment means were identified using Fisher’s protected Least Significant Difference test (*P* ≤ 0.05).

## Results

### Greenhouse trials

Results varied across the four greenhouse trials conducted in 2018 and 2019, so each trial was analyzed independently. Significant differences in root galling were found in all four trials. Galling was lowest on entries CC 13 and STNCB 2-28 in every experiment, and highest on the susceptible entry Hicks in all but the April–May 2018 trial (Table 1). Galling of CC 13 and STNCB 2-28 was significantly less than that of other entries in all tests except the April–May 2018 trial, and in the trial conducted in 2019, in which galling of CC 13 was similar to that of K326. Galling was significantly lower on CC 13 and STNCB 2-28 compared to entries K326 and T-15-1-1, which respectively possess *Rk1* and *Rk2* alone, in all studies but the April–May 2018 trial; in this trial, galling of K326 was similar to that of CC 13 and STNCB 2-28. Galling was significantly lower on entry K326 than T-15-1-1 in two of the four trials, and galling of both of these entries was significantly lower than that of Hicks in two trials. However, in the trial conducted in Blackstone from April to May of 2018, galling of Hicks was significantly less than that of T-15-1-1.

**Table 1.** Root galling of flue-cured tobacco entries by *Meloidogyne arenaria* race 2 in greenhouse pot tests in 2018 and 2019.^a^

		Root Galling (0–5)^b^			
		Blackstone			Blacksburg
		2019	2018		2018
Genotype	Entry	April–May	April–May	September	September
*rk1rk2*	Hicks	4.7 a	3.6 b	5.0 a	4.7 a
*RK1rk2*	K326	2.8 c	3.1 b	3.4 b	4.4 a
*rk1RK2*	T-15-1-1	3.5 b	4.4 a	3.9 b	4.6 a
*RK1RK2*	CC 13	2.3 cd	2.6 b	2.1 c	3.0 b
*RK1RK2*	STNCB 2-28	1.8 d	3.1 b	2.1 c	2.6 b

a Data presented are non-transformed means from seven, seven, six and seven replications respectively, inoculated with 5,000 *M. arenaria* eggs. Data were transformed (log_10_ + 1) prior to analysis of variance. Means within a column followed by the same letter(s) are not significantly different according to Fisher’s LSD test (*P* ≤ 0.05).

b Taylor and Sassers’ Indexed Scale of Gall Count-0 = 0; 1 = 1 to 2; 2 = 3 to 10; 3 = 11 to 30; 4 = 31 to 100; and 5 = > 100 galls per root system.

Nematodes per gram of root varied considerably among trials, but were typically lowest on CC 13 and/or STNCB 2-28 (Table 2). Roots of CC 13 and STNCB 2-28 always contained significantly fewer nematodes than susceptible entry Hicks, except in the April–May trial conducted in 2018, in which CC 13 was the only entry with significantly fewer nematodes in roots relative to the other four entries. Significantly fewer nematodes were present in the roots of both K326 and T-15-1-1 than Hicks in the trial conducted in Blackstone in September of 2018, and K326 had fewer nematodes in roots than Hicks in the trial conducted in 2019. Significantly fewer nematodes were present in the roots of entries CC 13 and STNCB 2-28 than entries K326 and T-15-1-1 in the trials conducted in both locations in September of 2018.

**Table 2.** Numbers of inoculated *Meloidogyne arenaria* race 2 nematodes observed in roots of flue-cured tobacco entries at the conclusion of greenhouse pot tests in 2018 and 2019.^a^

		Nematodes/g feeder root			
		Blackstone			Blacksburg
		2019	2018		2018
Genotype	Entry	April–May	April–May	September	September
*rk1rk2*	Hicks	84 a	55 a	26 a	144 a
*RK1rk2*	K326	25 bc	28 a	10 b	169 a
*rk1RK2*	T-15-1-1	50 ab	47 a	9 b	158 a
*RK1RK2*	CC 13	13 cd	14 b	6 c	69 b
*RK1RK2*	STNCB 2-28	11 d	26 a	3 c	45 b

a Data presented are non-transformed means from seven, seven, six and seven replications respectively, inoculated with 5,000 *M. arenaria* eggs. Data were transformed (log_10_ + 1) prior to analysis of variance. Means within a column followed by the same letter(s) are not significantly different according to Fisher’s LSD test (*P* ≤ 0.05).

Egg mass counts differed significantly in all trials. CC 13 and STNCB 2-28 always had significantly fewer egg masses compared to Hicks, and less than K326 in three trials (Table 3). Egg mass production was lower on both CC 13 and STNCB 2-28 than T-15-1-1 in one trial, although in another trial, CC 13 had significantly fewer egg masses than T-15-1-1, while egg mass production on STNCB 2-28 was intermediate. Significantly fewer egg masses were present on T-15-1-1 relative to Hicks in two trials, and in only one trial did K326 exhibit significantly lower egg mass production than Hicks.

**Table 3.** Egg masses, eggs per gram of root, and reproductive indices of *Meloidogyne arenaria* race 2 on flue-cured tobacco entries from greenhouse pot tests in 2018 and 2019.^a^

		Blackstone			Blacksburg
		2019	2018		2018
Genotype	Entry	April–May	April–May	September	September
*Egg masses per gram of root*					
*rk1rk2*	Hicks	7 a	25 a	2 a	52 ab
*RK1rk2*	K326	4 a	6 bc	1 a	60 a
*rk1RK2*	T-15-1-1	1 b	11 ab	0 b	32 b
*RK1RK2*	CC 13	1 b	3 c	0 b	20 c
*RK1RK2*	STNCB 2-28	1 b	6 bc	0 b	13 c
*Eggs per gram of root*					
*rk1rk2*	Hicks	30 a	754 a	1 b	1,038 a
*RK1rk2*	K326	42 a	60 b	10 ab	1,182 a
*rk1RK2*	T-15-1-1	8 a	215 ab	2 b	795 a
*RK1RK2*	CC 13	37 a	53 b	22 a	241 b
*RK1RK2*	STNCB 2-28	10 a	96 b	4 ab	264 b
*Reproductive index* ^*b*^					
*rk1rk2*	Hicks	0.1 a	2.2 a	0.0 a	2.4 a
*RK1rk2*	K326	0.1 a	0.3 b	0.0 a	2.3 a
*rk1RK2*	T-15-1-1	0.0 a	0.8 b	0.0 a	1.4 b
*RK1RK2*	CC 13	0.0 a	0.2 b	0.0 a	0.5 c
*RK1RK2*	STNCB 2-28	0.1 a	0.2 b	0.0 a	0.4 c

a Data presented are non-transformed means from seven, seven, six and seven replications respectively, inoculated with 5,000 *M. arenaria* eggs. Data were transformed (log_10_ + 1) prior to analysis of variance. Means within a column followed by the same letter(s) are not significantly different according to Fisher’s LSD test (*P* ≤ 0.05).

b Reproductive index = final population/initial population (P_f_/P_i_).

Reproduction varied dramatically across trials. Egg production was significantly lower on CC 13 and STNCB 2-28 than on all other entries in the trial conducted in September of 2018 in Blacksburg, and along with K326, was less than that of Hicks in the trial conducted from April to May in 2018 in Blackstone (Table 3). Egg counts were low (not exceeding 22 eggs per gram of root) across all entries in the trial conducted in September of 2018 in Blackstone, but both Hicks and T-15-1-1 had significantly fewer eggs per gram of root than CC 13 in this trial. There was no consistent trend in relative egg production among the susceptible entry and those possessing either *Rk1* or *Rk2* alone in the three trials where significant differences were present. No differences in egg production were found among entries in the trial conducted from April to May of 2019 in Blackstone, in which egg counts were also relatively low.

Reproductive indices for all four entries possessing *Rk1* and/or *Rk2* were significantly lower than that of susceptible Hicks in the trial conducted April to May of 2018 in Blackstone (Table 3). In the trial conducted in Blacksburg in September of 2018, the reproductive indices of T-15-1-1, CC 13, and STNCB 2-28 were significantly lower than those of Hicks and K326, while CC 13 and STNCB 2-28 were also significantly lower than T-15-1-1. In the remaining trials, reproduction was low and significant differences in reproductive indices were not detected (Table 3).

### Field trials

No root galling was observed on the roots of plants sampled at 3 and 6 weeks after transplanting in 2018 (Table 4). Limited galling was observed on the root systems (less than 10 galls per root system) of all entries at 9 and 12 weeks, but no significant differences were found. Galling of STNCB 2-28 was significantly less than that of Hicks, K326, and T-15-1-1 at 15 weeks, and was significantly less than that of K326 at 18 weeks.

**Table 4.** Root galling by *Meloidogyne arenaria* race 2 on flue-cured tobacco entries in field trials in Palmer Springs, VA in 2018 and 2019.^a^

		Root galling (0–5)^b^					
		Weeks after transplanting					
Genotype	Entry	3	6	9	12	15	18
*2018*							
*rk1rk2*	Hicks	0.0 a	0.0 a	0.8 a	0.9 a	1.9 a	2.7 ab
*RK1rk2*	K326	0.0 a	0.0 a	0.9 a	1.2 a	1.8 a	3.4 a
*rk1RK2*	T-15-1-1	0.0 a	0.0 a	0.6 a	1.2 a	1.9 a	2.1 ab
*RK1RK2*	CC 13	0.0 a	0.0 a	0.9 a	0.8 a	1.1 ab	2.5 ab
*RK1RK2*	STNCB 2-28	0.0 a	0.0 a	0.8 a	0.4 a	0.2 b	1.5 b
*2019*							
*rk1rk2*	Hicks	0.1 a	0.4 a	2.0 abc	2.0 a	2.0 a	2.4 a
*RK1rk2*	K326	0.1 a	0.4 a	2.6 ab	2.3 a	2.0 a	2.6 a
*rk1RK2*	T-15-1-1	0.1 a	0.6 a	3.6 a	1.3 ab	0.7 a	2.6 a
*RK1RK2*	CC 13	0.2 a	0.0 a	0.4 c	0.5 b	1.3 a	1.8 ab
*RK1RK2*	STNCB 2-28	0.1 a	0.3 a	0.8 bc	0.3 b	2.0 a	0.8 b

a Data presented are non-transformed means from eleven, eleven, eight, ten, seven, and eleven replications respectively in 2018, and ten, seven, four, six, three, and four replications respectively in 2019. Data were transformed (log_10_ + 1) prior to analysis of variance. Means within a column followed by the same letter(s) are not significantly different according to Fisher’s LSD test (*P* ≤ 0.05).

b Taylor and Sassers’ Indexed Scale of Gall Count-0 = 0; 1 = 1 to 2; 2 = 3 to 10; 3 = 11 to 30; 4 = 31 to 100; and 5 = > 100 galls per root system.

In 2019, galling was observed on the roots of plants sampled at 3 and 6 weeks after transplanting, but no significant differences were found among entries; no more than one or two galls were observed on root systems (Table 4). CC 13 and STNCB 2-28 both had significantly less galling than T-15-1-1 at 9 weeks, and at this timepoint galling of CC 13 was also significantly less than that of entry K326, while galling of susceptible Hicks was intermediate. Galling was significantly less on both CC 13 and STNCB 2-28 relative to K326 and Hicks at 12 weeks after transplanting, while at 18 weeks, STNCB 2-28 had significantly less root-galling than Hicks, K326 and T-15-1-1; no significant differences were observed at 15 weeks.

Significantly fewer nematodes were present in the roots of T-15-1-1 than CC 13 3 weeks after transplanting in 2018, whereas in 2019, fewer nematodes were present in the roots of CC 13 than Hicks, K326, and T-15-1-1 (Table 5). No differences in nematodes abundance were found at 6 weeks in either year, and in 2018, no significant differences were present at 9 weeks. In 2019, CC 13 had significantly fewer nematodes present in roots than K326. In both years, starting at 12 weeks, STNCB 2-28 had the fewest nematodes in roots; with the exception of 15 weeks in 2018, STNCB 2-28 always had significantly fewer nematodes present in roots than Hicks, and often relative to K326 as well, while other entries were typically intermediate in nematode abundance. The number of nematodes present in the roots of entry CC 13, which is homozygous for *Rk1* and heterozygous for *Rk2*, was never significantly different from that of Hicks.

**Table 5.** Numbers of *Meloidogyne arenaria* race 2 nematodes observed in roots of flue-cured tobacco entries at six timepoints from field trials in Palmer Springs, VA in 2018 and 2019.^a^

		Nematodes/g root					
		Weeks after transplanting					
Genotype	Entry	3	6	9	12	15	18
2018							
*rk1rk2*	Hicks	1 ab	5 a	47 a	84 a	35 ab	38 a
*RK1rk2*	K326	2 ab	3 a	25 a	73 ab	32 ab	62 a
*rk1RK2*	T-15-1-1	1 b	7 a	27 a	49 ab	40 a	31 ab
*RK1RK2*	CC 13	3 a	5 a	7 a	63 a	11 ab	46 a
*RK1RK2*	STNCB 2-28	1 ab	3 a	31 a	33 b	8 b	16 b
*2019*							
*rk1rk2*	Hicks	5 a	9 a	20 ab	55 a	35 a	37 a
*RK1rk2*	K326	7 a	4 a	26 a	79 a	41 a	32 ab
*rk1RK2*	T-15-1-1	5 a	14 a	13 ab	20 ab	8 ab	14 ab
*RK1RK2*	CC 13	2 b	4 a	5 b	39 a	18 ab	16 ab
*RK1RK2*	STNCB 2-28	5 ab	4 a	13 ab	6 b	5 b	6 b

a Data presented are non-transformed means from eleven, eleven, seven, nine, seven, and ten replications respectively in 2018, and ten, seven, four, six, three, and four replications respectively in 2019. Data were transformed (log_10_ + 1) prior to analysis of variance. Means within a column followed by the same letter(s) are not significantly different according to Fisher’s LSD test (*P* ≤ 0.05).

Egg masses and/or eggs were not observed on any entry at 3 or 6 weeks after transplanting in either year, and in both years no significant differences were observed at 9 weeks after transplanting, when reproduction was first observed (Table 6). In both years, STNCB 2-28 typically had the fewest egg masses present, although there were notable exceptions to this trend, particularly at 12 weeks in 2018, when K326 had significantly fewer egg masses present than Hicks, while all other entries experienced intermediate egg mass production. No single entry consistently exhibited the highest egg mass numbers in either year. In 2019, egg production on STNCB 2-28 was significantly less than on K326, while no significant differences were found at this timepoint in 2018. At 15 weeks in 2018, egg production was significantly lower on CC 13, STNCB 2-28, and susceptible Hicks relative to T-15-1-1, whereas at the same timepoint in 2019, egg counts were significantly lower for CC 13 and STNCB 2-28 relative to Hicks. No significant differences in egg production were found at 18 weeks in either year.

**Table 6.** Egg mass and egg production by *Meloidogyne arenaria* race 2 on flue-cured tobacco entries in field trials in Palmer Springs, VA in 2018 and 2019.^a^

		Weeks after transplanting			
Genotype	Entry	9	12	15	18
*Egg masses per gram of root*					
2018					
*rk1rk2*	Hicks	2 a	38 a	8 ab	16 ab
*RK1rk2*	K326	0 a	1 b	1 b	21 a
*rk1RK2*	T-15-1-1	1 a	11 ab	16 a	19 ab
*RK1RK2*	CC 13	0 a	24 ab	4 b	21 ab
*RK1RK2*	STNCB 2-28	0 a	28 ab	1 b	6 b
*2019*					
*rk1rk2*	Hicks	3 a	14 a	31 a	27 a
*RK1rk2*	K326	5 a	34 a	39 a	26 ab
*rk1RK2*	T-15-1-1	4 a	18 a	5 a	4 b
*RK1RK2*	CC 13	2 a	14 ab	11 a	9 ab
*RK1RK2*	STNCB 2-28	2 a	1 b	0 a	5 b
*Eggs per gram of root*					
2018					
*rk1rk2*	Hicks	28 a	212 a	38 b	80 a
*RK1rk2*	K326	50 a	225 a	45 ab	133 a
*rk1RK2*	T-15-1-1	27 a	105 a	168 a	79 a
*RK1RK2*	CC 13	22 a	266 a	43 b	95 a
*RK1RK2*	STNCB 2-28	48 a	445 a	39 b	50 a
2019					
*rk1rk2*	Hicks	684 a	3,885 ab	12,457 a	2,328 a
*RK1rk2*	K326	473 a	7,686 a	4,673 ab	2,276 a
*rk1RK2*	T-15-1-1	1,284 a	3,830 ab	2,223 ab	995 a
*RK1RK2*	CC 13	643 a	3,120 ab	989 b	1,347 a
*RK1RK2*	STNCB 2-28	1,129 a	1,296 b	1,231 b	793 a

a Data presented are non-transformed means from eleven, eleven, seven, nine, seven, and ten replications respectively in 2018, and ten, seven, four, six, three, and four replications respectively in 2019. Data were transformed (log_10_ + 1) prior to analysis of variance. Means within a column followed by the same letter(s) are not significantly different according to Fisher’s LSD test (*P* ≤ 0.05).

Reproductive indices were not calculated for field trials because the amount of egg inoculum present in the soil at transplanting can only be speculated upon based on pre-plant root-knot second-stage juvenile abundance. Mean initial and final soil *M. arenaria* counts are presented in Table 7. In our 2018 study, *M. arenaria* juveniles were not present at detectable levels in pre-plant soil samples from 71% of the plots, while following termination of the study, *M. arenaria* juveniles were not present at detectable levels in soil nematode extracts from 10% of the studied plots. In 2019, *M. arenaria* juveniles were not present at detectable levels in pre-plant soil nematode extracts from 53% of plots, while following termination of the 2019 study, *M. arenaria* juveniles were not present at detectable levels in soil nematode extracts from 56% of the studied plots.

**Table 7.** Initial and finial soil nematode counts for flue-cured tobacco entries in field trials in Palmer Springs, VA in 2018 and 2019.^a^

		*Meloidognye arenaria* juveniles/500 cc of soil	
Genotype	Entry	Before planting	After final harvest
*2018*			
*rk1rk2*			
*RK1rk2*	K326	13	2,753
*rk1RK2*	T-15-1-1	22	1,858
*RK1RK2*	CC 13	29	1,382
*RK1RK2*	STNCB 2-28	11	753
*2019*			
*rk1rk2*	Hicks	64	8
*RK1rk2*	K326	12	96
*rk1RK2*	T-15-1-1	60	24
*RK1RK2*	CC 13	26	44
*RK1RK2*	STNCB 2-28	50	12

a Data presented are non-transformed means from eleven and ten replications respectively in 2018, and ten and four replications respectively in 2019.

## Discussion

Results of our greenhouse trials suggest that the presence of both resistance genes *Rk1* and *Rk2* increases resistance to *M. arenaria* race 2 in flue-cured tobacco relative to susceptible entries and those possessing either resistance gene alone. These results confirm findings of previous studies demonstrating that a combination of both *Rk1* and *Rk2* increases resistance to root-knot nematodes more than either gene alone, effective against *M. javanica* (Ternouth et al., 1986; Ma et al., unpublished data), *M. incognita* races 1 and 3 (Barker and Melton, 1990; Ng’ambi et al., 1999a, b; Pollock et al., 2016) and *M. arenaria* races 1 and 2 (Ng’ambi et al., 1999a, b; Pollok et al., 2015).

Our results also suggest that the zygosity of *Rk2* when present in combination with the homozygous *Rk1* gene does not have a significant effect on root galling and root-knot nematode reproduction. In our greenhouse trials, we only observed one case in which root-knot parasitism differed significantly between entry CC 13, which is homozygous for *Rk1* and heterozygous for *Rk2*, and STNCB 2-28, which is homozygous for both genes. In that one case, CC 13 actually exhibited fewer nematodes in roots than STNCB 2-28 (in the trial conducted from April to May in Blackstone). Significantly fewer nematodes were present in the roots of STNCB 2-28 than CC 13 at three timepoints in our field trials (12 weeks after transplanting in both 2018 and 2019, and 18 weeks in 2018). However, we never found significant differences between these two entries in root galling or nematode reproduction, again suggesting that the heterozygosity of *Rk2* in combination with *Rk1* may not adversely impact nematode resistance of flue-cured tobacco under field conditions. In a field trial conducted at the same location in 2014, galling of entries possessing both *Rk1* and *Rk2* was significantly less than entries possessing either gene alone and a susceptible check (Pollok et al., 2015).

The relationship between the presence of either resistance gene alone and relative inhibition of nematode parasitism by *M. arenaria* race 2 was somewhat less clear in our greenhouse data, but suggests that *Rk2* may be somewhat more effective against *M. arenaria* race 2 than *Rk1*. The number of nematodes present in roots never differed between entries K326, which possesses *Rk1* only, and T-15-1-1 (possessing only *Rk2*), but root galling was significantly lower on T-15-1-1 than K326 in two of four trials. While the number of egg masses were significantly lower for T-15-1-1 versus K326 in three of our trials, egg counts and reproductive indices were similar on these entries in all trials, except in the trials conducted in September of 2018 in Blackstone, when the reproductive index on T-15-1-1 was lower than on K326. Under field conditions, the number of nematodes present in roots, root galling, and egg production were not significantly different at any timepoint in either year.

Nematode inoculum was clearly viable in two of our four greenhouse trials as assessed by penetration and gall index, but reproduction was very low (reproductive indices not exceeding 0.1) and no significant differences could be found among any entries. This is not entirely surprising given that the root-knot nematode lifecycle typically takes about 25 days at 27°C (Agrios, 2005), varying around this average based on a number of factors including host plant, root-knot species and environmental conditions, and temperature in particular (Eisenback and Triantaphyllou, 1991). These trials were both conducted in the Spring and Fall of 2019 and 2018, respectively, in the same greenhouse in Blackstone. However, a concurrent study was conducted with inoculum from the same population of *M. arenaria* race 2 in Blacksburg in the Fall of 2018, in which reproduction was relatively high (reproductive indices ranged from 0.5 to 2.4). We speculate that some difference in temperature or the timing of the removal of the shade cloths from the two greenhouse facilities may account for this discrepancy between the two trials conducted in the Fall of 2018, and the generally low reproduction in the two relevant trials.

The results from our field trials were unclear, with few significant differences found among entries in either year. Root-knot nematode pressure in the field was highly variable in both years and probably accounted for substantial variability in our results. In addition, sampling resolution remains an ongoing issue; pre- and post-season soil nematode counts found few or no root-knot nematode juveniles in many plots where plants did in fact experience severe nematode parasitism; the opposite was also true for many plots in which many root-knot nematodes were found in pre- and post-season counts. Novel sampling methods, randomization philosophies and analytical techniques have been developed recently to address similar issues in field trials assessing host resistance and tolerance to root lesion nematodes (*Pratylenchus* spp.) in Australia (Reeves et al., 2020) that may offer solutions in future field trials assessing host resistance to root-knot nematodes in tobacco. Using these methods, treatments may be randomized in a manner that exposes all treatments to similar nematode densities while also retaining statistical power.

The results of our study suggest *Rk1* reduces nematode feeding site initiation by *M. arenaria* race 2, but subsequent reductions in fecundity were not significant relative to a susceptible host. We observed significant reductions in galling on K 326, which possesses *Rk1* only, relative to the susceptible control Hicks in two of four trials, but significant reductions in nematode reproduction on K 326 versus Hicks were only observed in one trial.

The mechanism of resistance associated with *Rk2* is not clear, nor is that afforded by the combination of *Rk1* with *Rk2* (Pollok et al., 2016). In a 1982 report, Shepherd described significant reductions in root penetration by *M. javanica* juveniles on ‘better breeding lines’ that were not identified specifically. However, subsequent development of the successful juveniles was not impacted by the trait possessed by these lines (Shepherd, 1982). Based on later reports from this research group (Ternouth et al., 1986), these ‘better breeding lines’ may have possessed the ‘*T*’ or *Rk2* trait, in which case the implied mechanism of resistance would be different from the hypersensitive response conferred by *Rk1*. Pollok et al. (2015) reported that *Rk2* did not significantly reduce galling by *M. arenaria* race 2 relative to a susceptible entry under field conditions, but reductions in galling were significant when *Rk2* was combined with *Rk1*. Pollok et al. (2016) also observed limited reductions in galling caused by *M. incognita* race 3 on plants possessing only *Rk2* in greenhouse trials. They also observed that subsequent nematode development was inhibited by *Rk2,* while the presence of both resistance genes reduced all metrics of root-knot nematode parasitism. In our study, *Rk2* alone reduced root galling compared to the susceptible control in two of four trials, and the number of penetrated nematodes present in roots was also significantly lower than in the susceptible entry in one of these trials. We observed significant reductions in reproduction associated with *Rk2,* as expressed by reproductive indices, in two trials; but, in no case were actual egg counts significantly reduced relative to the susceptible entry. Thus, taken along with the results presented by other authors, our results do not necessarily clarify the mode of action associated with *Rk2*. Similarly, while our results confirm that the presence of both genes imparts increased resistance to root-knot nematodes, the specific mechanism of inhibition associated with this stacked resistance remains unclear.

Despite the variability observed in our trials, our results suggest that commercially available tobacco cultivars possess partial resistance to all of the most widely distributed, historically important, root-knot nematode parasites of flue-cured tobacco. However, the increasing abundance of *M. enterolobii* in flue-cured tobacco production regions in the southeastern United States presents a new root-knot nematode pest challenge that cannot be mitigated by currently available forms of host resistance, including genes of the *Rk* and *Mi* families (Ye, 2018). Further research is necessary to identify sources of resistance to this new nematode threat, which could displace other species and present tobacco growers with a pest that cannot be managed with commercially available host resistance.

## References

[ref001] Agrios, G. N. 2005. “Plant diseases caused by nematodes”, In Agrios, G. N. (Ed.), Plant Pathology 5th ed., Academic Press, Cambridge, pp. 825–874.

[ref002] Barker, K. R. 1985. “Nematode extraction and bioassays”, In Barker, K. R., Carter, C. C. and Sasser, J. N. (Eds), An Advanced Treatise on *Meloidogyne *Vol. II: Methodology Department of Plant Pathology, North Carolina State University and U.S Agency for International Development, Raleigh, NC, pp. 19–35.

[ref003] Barker, K. R. and Melton, T. A. 1990. Comparative host sensitivity and efficacy of selected tobacco cultivars to *Meloidogyne* species and populations. Tobacco Science 34:44–49.

[ref004] Byrd, D. W. Jr , Kirkpatrick, T. and Barker, K. R. 1983. An improved technique for clearing and staining plant tissues for detection of nematodes. Journal of Nematology 15:142–143. 19295781 PMC2618249

[ref005] Daykin, M. E. and Hussey, R. S. 1985. “Staining and histopathological techniques in nematology”, In Barker, K. R., Carter, C. C. and Sasser, J. N. (Eds), An Advanced Treatise on *Meloidogyne* Vol. II: Methodology Department of Plant Pathology, North Carolina State University and U.S Agency for International Development., Raleigh, N.C, p. 41.

[ref006] Eisenback, J. D., 1985. Techniques for preparing nematodes for scanning electron microscopy”, In Barker, K. R., Carter, C. C. and Sasser, J. N. (Eds), An Advanced Treatise on *Meloidogyne*, vol. II, Methodology North Carolina State University Graphics, Raleigh, NC, pp. 79–105.

[ref007] Eisenback, J. D. 2012. Effects of resistant tobacco on population dynamics of root-knot nematode species in Virginia. 45th Tobacco Workers Conference, Williamsburg, VA, Paper 89 (Abstract) January, pp. 16–19.

[ref008] Eisenback, J. D. and Triantaphyllou, H. H. 1991. “Root-knot nematodes: *Meloidogyne* species and races”, In Nickle, W. R. (Ed.), Manual of Agricultural Nematology Marcell Dekker, New York, NY, pp. 191–274.

[ref009] FAO 2016. FaoStat Database Collections, available from: http://www.fao.org/faostat/en/?#data/QV (accessed September 15, 2020).

[ref010] Fortnum, B. A., Lewis, S. A. and Johnson, A. W. 2001. Crop rotation and nematicides for management of mixed populations of *Meloidogyne* spp. on tobacco. Journal of Nematology 33:318–324. 19265896 PMC2620523

[ref011] Hussey, R. S. and Barker, K. R. 1973. A comparison of methods of collecting inocula of *Meloidogyne *spp. including a new technique. Plant Disease Reporter 57:1025–1028.

[ref012] Jack, A. M. 2001. Kutsaga RK26 – a new root-knot, Granville wilt and black shank resistant variety. Zimbabwe Tobacco 10:25–31.

[ref013] Jack, A. M. and Lyle, J. 1999. Kutsaga RK26 – a new root-knot resistant variety. Zimbabwe Tobacco 8:28–31.

[ref014] Johnson, C. S. 1989. Managing root-knot on tobacco in the southeastern United States. Journal of Nematology 21:604. 19287655 PMC2618998

[ref015] Johnson, C. S. 2020. “Disease control”, In Reed, T. D., Johnson, C. S., Wilkinson, C. A. and Barts, S. (Eds), 2020 flue-cured Tobacco Production Guide 436-048 VA Cooperative Extension Publication, Blacksburg, VA, pp. 47–59.

[ref016] Johnson, C. S., Way, J. and Barker, K. R. 2005. “Nematode parasites of tobacco”, In Luc, M., Sikora, R. A. and Bridge, J. (Eds), Plant Parasitic Nematodes in Subtropical and Tropical Agriculture 2nd ed, CAB International, Wallingford, pp. 675–708.

[ref018] Koenning, S. R., Overstreet, C., Noling, J. W., Donald, P. A., Becker, J. O. and Fortnum, B. A. 1999. Survey of crop losses in response to phytoparasitic nematodes in the United States for 1994. Journal of Nematology 31:587–618. 19270925 PMC2620402

[ref019] LaMondia, J. A. 2008. Early crop root destruction for management of tobacco cyst nematodes. Journal of Nematology 40:26–29. 19259515 PMC2586525

[ref020] Mackenzie, J., Smeeton, B. W., Jack, A. M. and Ternouth, R. A. F. 1986. “Review on breeding for resistance to root-knot, *Meloidogyne javanica*, in flue-cured tobacco in Zimbabwe”, Proceedings of CORESTA Symposium, Taormina, Sicily, October 26–30, pp. 272–6.

[ref021] Ng’ambi, T. B. S., Rufty, R. C. and Barker, K. R. 1999a. Genetic analysis of *Meloidogyne arenaria* Race 1 resistance in tobacco. Plant Disease 83:810–813. 30841036 10.1094/PDIS.1999.83.9.810

[ref022] Ng’ambi, T. B. S., Rufty, R. C., Barker, K. R. and Melton, T. A. 1999b. Identification of sources of resistance to four species of root-knot nematodes in tobacco. Journal of Nematology 31:272–282. 19270897 PMC2620380

[ref023] Pollok, J. R., Darnell, L. A., Johnson, C. S. and Reed, T. D. 2015. Resistance to root-knot nematode in flue-cured tobacco cultivars in Virginia, 2014. Plant Disease Management Report 9:N015.

[ref024] Pollok, J. R., Johnson, C. S., Eisenback, J. D. and Reed, T. D. 2016. Reproduction of *Meloidogyne * *incognita* race 3 on flue-cured tobacco homozygous for Rk1 and/or Rk2 resistance genes. Journal of Nematology 2:79–86. 10.21307/jofnem-2017-012 PMC4930319 27418700

[ref025] Reed, T. D. 2007. “Flue-cured tobacco disease control”, In Reed, T. D., Johnson, C. S., Semtner, P. J. and Wilkinson, C. A. (Eds), 2007 Flue-cured Tobacco Production Guide 436-048 VA Cooperative Extension Publication, Blacksburg, VA, pp. 5–36.

[ref026] Reed, T. D., Johnson, C. S., Semtner, P. J. and Wilkinson, C. A. 2018. 2018 Flue-cured tobacco production guide Va. Coop. Ext. Publ. No. 436-048, Blacksburg, VA.

[ref027] Reeves, K. L., Forknall, C. R., Kelly, A. M., Owen, K. J., Fanning, J., Hollaway, G. J. and Loughman, R. 2020. A novel approach to the design and analysis of field experiments to study variation in the tolerance and resistance of cultivars to root lesion nematodes (*Pratylenchus* spp.). Phytopathology 110:1623–1631. 32479206 10.1094/PHYTO-03-20-0077-R

[ref028] Roberts, P. A. 2002. “Concepts and consequences of resistance”, In Starr, J. L., Cook, R. and Bridge, J. (Eds), Plant Resistance to Parasitic Nematodes CAB International, Wallingford, pp. 23–41.

[ref029] Schneider, S. M. 1991. Penetration of susceptible and resistant tobacco cultivars by *Meloidogyne * *juveniles*. Journal of Nematology 23:225–228. 19283116 PMC2619151

[ref030] Schweppenhauser, M. A. 1975. A source of *Nicotiana tabacum* resistant to *Meloidogyne *j *avanica*. Tobacco Science 19:42–45.

[ref031] Shepherd, J. A. 1982. Report to the third regional conference on root-knot nematode research held at the international institute of tropical agriculture, Ibidan, Nigeria. Proceedings of the 3rd Research Planning Conference on root-knot nematodes, *Meloidogyne* spp., Regions IV and V. November 16–20, 1981.

[ref032] Taylor, A. L. and Sasser, J. N. 1978. Identification and control of root-knot nematodes (*Meloidogyne* spp.) Crop Publ. Department of Plant Pathology, North Carolina State University and U.S Agency for International Development, Raleigh, NC, p. 111.

[ref033] Ternouth, R. A. F., Mackenzie, J. and Shepherd, J. A. 1986. “Introduction of *Meloidogyne javanica* resistance into flue-cured tobacco in Zimbabwe”, Proc. CORESTA Symposium, Taormina, Sicily, October 26–30, pp. 277–80.

[ref034] USDA 2020. United States Department of Agriculture, National Agricultural Statistics Service, Acreage, June, available at: www.nass.usda.gov/publications/reports/acrg0620 (accessed November 16, 2020).

[ref035] USEPA 2008. United States Environmental Protection Agency, implementation of risk mitigation measures for soil fumigant pesticides, available at: http://www.epa.gov/pesticides/reregistration/soil_fumigants/ (accessed November 22, 2020).

[ref036] Way, J. I. 1994. “Susceptibility of four tobacco cultivars to *Meloidogyne* species”, CORESTA Information Bulletin, Congress Issue, Harare, Zimbabwe, October 9–24, p. 110 (Abstract).

[ref037] Ye, W. 2018. “Nematodes of agricultural importance in North and South Carolina”, In Chitambar, J. J. and Subbotin, S. A. (Eds), Plant Parasitic Nematodes in Sustainable Agriculture of North America Springer, New York, NY, pp. 247–276.

[ref038] Yi, H. Y., Rufty, R. C., Wernsman, E. A. and Conkling, M. C. 1998. Mapping the root-knot nematode resistance gene (*Rk*) in tobacco with RAPD markers. Plant Disease 82:1319–1322. 30845463 10.1094/PDIS.1998.82.12.1319

